# Convenient Access to Expert-Reviewed Health Information via an Alexa Voice Assistant Skill for Patients With Multiple Myeloma: Development Study

**DOI:** 10.2196/35500

**Published:** 2022-06-09

**Authors:** Marc-Andrea Baertsch, Sarah Decker, Leona Probst, Stefan Joneleit, Hans Salwender, Franziska Frommann, Hartwig Buettner

**Affiliations:** 1 Department of Internal Medicine V University of Heidelberg Heidelberg Germany; 2 Takeda Pharma Vertrieb GmbH & Co KG Berlin Germany; 3 Department of Hematology-Oncology Asklepios Tumorzentrum Hamburg Asklepios Klinik Altona and Asklepios Klinik St Georg Hamburg Germany; 4 Medizinisches Versorgungszentrum für Blut- und Krebserkrankungen Potsdam Germany

**Keywords:** Alexa voice assistant, Alexa, voice assistant, virtual assistant, multiple myeloma, cancer, oncology, medical education, patient support group, digital health, patient support, support group, Europe, German, mobile phone

## Abstract

**Background:**

Patients with multiple myeloma (MM) have high information needs due to the complexity of the disease and variety of treatments. Digital voice assistants provide support in daily life and can be a convenient tool that even older patients can use to access health information. Voice assistants may therefore be useful in providing digital health services to meet the information needs of patients with MM.

**Objective:**

We aim to describe and report on the development, content, and functionality of the first Amazon Alexa voice assistant skill for patients with MM in Germany with the goal of empowering and educating patients. Further, we share data on skill usage and first learnings.

**Methods:**

In a cocreation workshop with MM patient organizations and MM medical experts in Germany, Takeda Oncology discussed the development and content of the Alexa skill *Multiple Myeloma*. Patient information on MM disease, diagnostics, and therapy was presented in a question-and-answer format, reviewed by experts, and programmed into the skill. Additionally, a search function for finding patient support groups within a perimeter of 200 km around the users and a myeloma quiz functionality with multiple-choice questions were integrated into the skill. Aggregated retrospective data on the total number of skill installations and skill usage were retrieved from an Amazon Alexa developer account, and a web-based patient survey was conducted on the Takeda Oncology website.

**Results:**

The Alexa skill *Multiple Myeloma* was launched in September 2019. It was available free of charge on the German Amazon Alexa skill store between September 2019 and March 2022 and could be used with devices featuring the Amazon Alexa voice assistant. Since the launch in September 2019 and up to July 2021, a total of 141 users have installed the skill. Between July 2020 and July 2021, a total of 189 skill sessions with 797 utterances were analyzed. The most popular inquiries were searches for patient support groups near the users (58/797, 7.3%), followed by inquiries about information on MM disease (53/797, 6.6%) and the quiz (43/797, 5.4%). The web-based survey on voice assistant usage and the feedback on the Alexa skill *Multiple Myeloma* were collected from 24 participants and showed that 46% (11/24) of participants would recommend the Alexa skill. Nonusers of voice assistants (11/24, 46%) stated that data protection concerns (7/11, 64%) and a lack of need (6/11, 55%) were the most important factors of not using voice assistants.

**Conclusions:**

The Alexa skill *Multiple Myeloma* offers patient-friendly and expert-reviewed answers and explanations for medical terms related to MM disease, diagnostics, and therapy, as well as connections to patient support groups and a quiz functionality. In the future, the skill can be extended with new content and functionalities, such as medication adherence support.

## Introduction

Multiple myeloma (MM) is a malignant disease that is characterized by the presence of monoclonal plasma cells in bone marrow [1]. It is the second most frequent hematologic malignancy and accounts for 2% of all cancers [2]. As MM is diagnosed at a median age of 69 years, MM typically affects older individuals [1]. Despite advances in diagnostics and treatment that have resulted in the more than doubling of the median overall survival rate, MM remains largely incurable [3]. Patients frequently survive ≥10 years with the disease but require repeated treatment courses and ultimately enter a refractory state. Further, significant morbidity, which limits patients’ quality of life, is related to MM-induced bone damage and impaired kidney function, anemia, and hypercalcemia, as well as treatment-related toxicities [1]. The awareness of being diagnosed with an incurable disease further adds to the heavy burden on psychological well-being [4].

The complexity of the disease; the plethora of diagnostic and follow-up tests; and the various treatment options, including autologous stem cell transplantation, have resulted in a high, continuous information need in affected patients [5-7]. Receiving a diagnosis of MM and being confronted with specialized medical terms can be very overwhelming, and patients, as well as their caregivers, are often unable to address all of their questions directly to a health care professional when they occur. In addition, they may also be concerned about asking “stupid” questions or wasting the time of health care providers (HCPs). Thus, there is a need for patients and their families to have easy access to basic and accurate information at the time they need it and not at their next formal appointment [6,8]. Patient brochures or information on the internet might not be easily accessible for all patients, trustful sources on the internet are not easy to select due to the overflow of search engine results upon information retrieval, and older patients might have difficulties with reading or using a computer. With regard to bridging the gap between information needs and easy access to validated information, voice assistants may play an important role [9].

The Amazon Alexa voice assistant offers education and support in daily life and a new opportunity for providing convenient access to health information that can be delivered in a patient’s home [10]. The assistant has the potential to reach patient populations, especially older patients and patients living in rural areas, who otherwise might not engage with education and support. Alexa skills are small programs that are similar to apps; people can use them to obtain validated and expert-reviewed content instead of searching for such content via search engines. Alexa skills can be used with devices featuring Amazon Alexa (eg, Amazon smart speakers or smart televisions [TVs]) or with any smartphone that has the Amazon Alexa app and the integrated Alexa voice assistant. In 2019, the United Kingdom’s National Health Service (NHS) announced a partnership with Amazon Alexa, which aims to provide reliable health information from the NHS website through voice-assisted technology. The partnership claims to be a “world first” and aims to aid patients, especially older patients and patients with blindness, who cannot easily search for advice on the internet. The NHS expects voice-assisted technology to reduce the pressure on the NHS and HCPs by providing information for common illnesses [11,12]. In Germany, several health insurance programs have started to launch Alexa skills. Techniker Krankenkasse (“TK health insurance”) offers meditation training, mindfulness training, and relaxation exercises with the Alexa skill *TK Smart Relax* [13,14]. Deutsche Angestellten-Krankenkasse (“DAK health insurance”) offers exercises for people with dementia via the Alexa skill *Erinnerungs-Coach* [15]. The Alexa skill *Knappschaft Babyglück* offers weekly information and health advice during pregnancy [16]. However, although some Alexa health skills are available, there are none for MM (or oncology) in Germany yet.

We therefore developed the first Alexa voice assistant skill for MM in collaboration with patient organizations and HCPs, with the aim of educating and empowering patients with MM and their families.

## Methods

### Ethical Considerations

No application for an ethics review board assessment was submitted. As the retrieval of Amazon Alexa usage data was covered by data protection regulations and the data were made available by Amazon only in aggregated form, no individual user data were analyzed.

### Participants

The participants included Takeda Oncology, MM patient organizations (Myelom Deutschland e.V., Leukaemiehilfe Rhein-Main [LHRM] e.V., and Arbeitsgemeinschaft Multiples Myelom [AMM]-Online), and MM medical experts (office-based and hospital-based experts).

### Procedure

In June 2019, a cocreation workshop with 3 patient organizations (Myelom Deutschland e.V., LHRM e.V., and AMM-Online) and MM medical experts was organized by the pharmaceutical company Takeda Pharma Vertrieb GmbH & Co. KG, Berlin, Germany, to discuss the development and content of the Alexa skill *Multiple Myeloma*. Patient information on MM disease, diagnostics, and therapy was presented in a question-and-answer format (Alexa intents), reviewed by experts, and then programmed into the skill. The Alexa skill is able to answer questions that vary in terms of wording but have the same intent as long as it recognizes similar keywords. For example, a user can ask ”what are myeloma cells” or “what do you know about plasma cells” and receive the same expert-reviewed answer for explaining that myeloma cells are abnormal plasma cells. In alignment with German laws on advertising health-related products and services (*Heilmittelwerbegesetz*) [17], no information on specific medications was added, and patient-friendly language was used. Changes in information on MM disease, diagnostics, and therapy, as well as new information (eg, MM-related information on COVID-19 pandemic), could be rapidly programmed into the skill as new intents at any time. Additionally, a search function for finding patient support groups within a perimeter of 200 km around the users, which is based on publicly available data from patient organizations, was integrated in the skill. Further, to enrich interactivity, an MM quiz functionality with multiple-choice questions was added. The Alexa skill *Multiple Myeloma* was launched in September 2019, and it was available at no cost on the German Amazon Alexa store between September 2019 and March 2022. It can be used on different devices, including smart speakers, smart TVs, and smartphones by using the Amazon Alexa app with the integrated Alexa voice assistant. The skill is activated with the easy and intuitive keywords *Alexa, start/open Multiple Myeloma*.

### Usage Data

Usage data were retrieved retrospectively and in aggregated form, in accordance with data protection standards, from an Amazon Alexa developer account. These data included the total number of skill installations (user enablements) since the skill’s launch, as well as the number of sessions and inquiries (utterances) that were conducted from July 1, 2020, through July 1, 2021, and the topics (intents) that were the most popular during the same time frame.

### Patient Survey

To collect data on users’ first experiences with the Alexa skill, a web-based patient survey was conducted on the Takeda Oncology website. The survey consisted of 6 questions regarding the usage of voice assistants and experiences with the Alexa skill *Multiple Myeloma*, which were answered in a multiple-choice or free-text format. The survey was promoted by Takeda and via patient organization communication channels. The results were analyzed by using descriptive statistics in Microsoft Excel.

### Promotion

By performing multichannel promotion via a Google AdWords campaign, web-based banners, print advertisements, and flyers, we aimed to reach as many patients as possible. A website with an educational video on how to use the skill should additionally empower and motivate patients to use the skill [18].

## Results

### Skill Features

**Figure 1:**
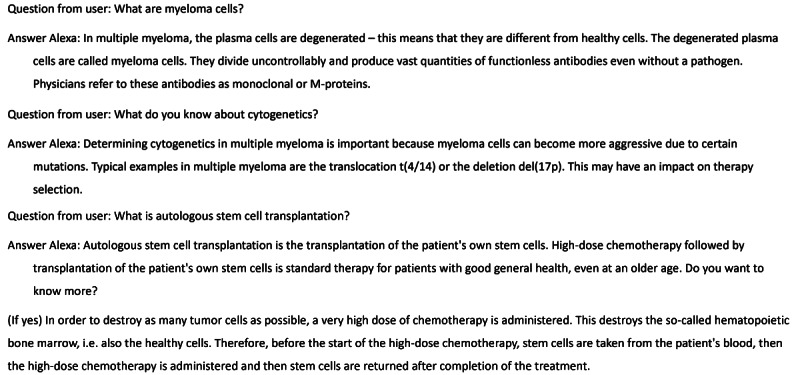
Examples of question-and-answer content from the Alexa skill “Multiple Myeloma." The original German content was translated for this publication.

The Alexa skill *Multiple Myeloma* was launched in September 2019 to empower and educate patients with MM and their families by offering easy access to validated information. The primary features of the skill are answering frequently asked questions and explaining medical terms related to MM disease, diagnostics, and therapy based on underlying expert-reviewed content. The skill currently contains over 30 intents for answering questions regarding MM disease, diagnostics, and therapy (Multimedia Appendix 1). With regard to myeloma disease, questions like “what are myeloma cells” and “what are typical symptoms” can be answered. Further, the skill answers questions regarding myeloma diagnosis, such as “how is myeloma diagnosed” and “what do you know about cytogenetics,” and questions regarding myeloma therapy, such as “how is myeloma treated,” ”why are combination therapies used,” and “what is a stem cell transplantation?” Sample questions and answers are shown in Figure 1.

**Figure 2:**
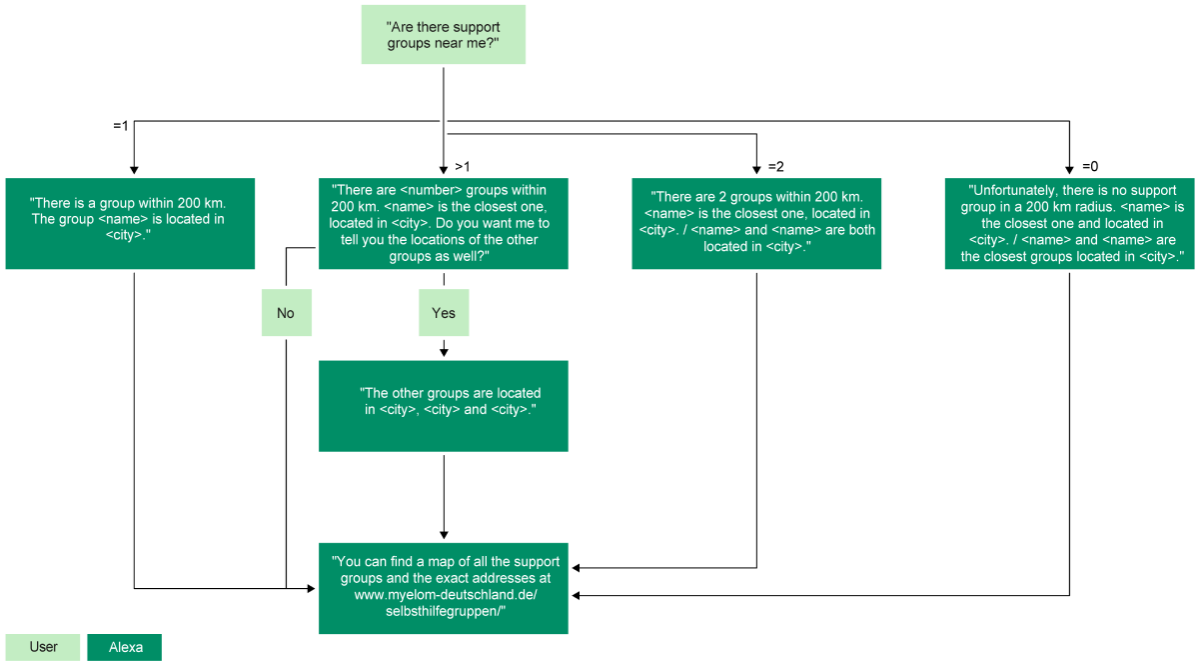
The algorithm of the search function for finding support groups for patients with multiple myeloma.

A search function for 29 local German support groups for patients with myeloma was integrated into the skill. This function searches for patient support groups within the perimeter of 200 km around the users (eg, “What patient support groups are near me?”) or in a specific city (eg, “Is there a patient support group in Berlin?”) based on publicly available data from patient organizations (Multimedia Appendix 1). The algorithm of the search function is shown in Figure 2.

**Figure 3:**
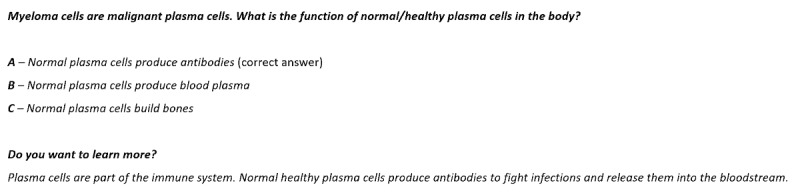
Sample question from the quiz in the Alexa skill “Multiple Myeloma”.

**Figure 4:**
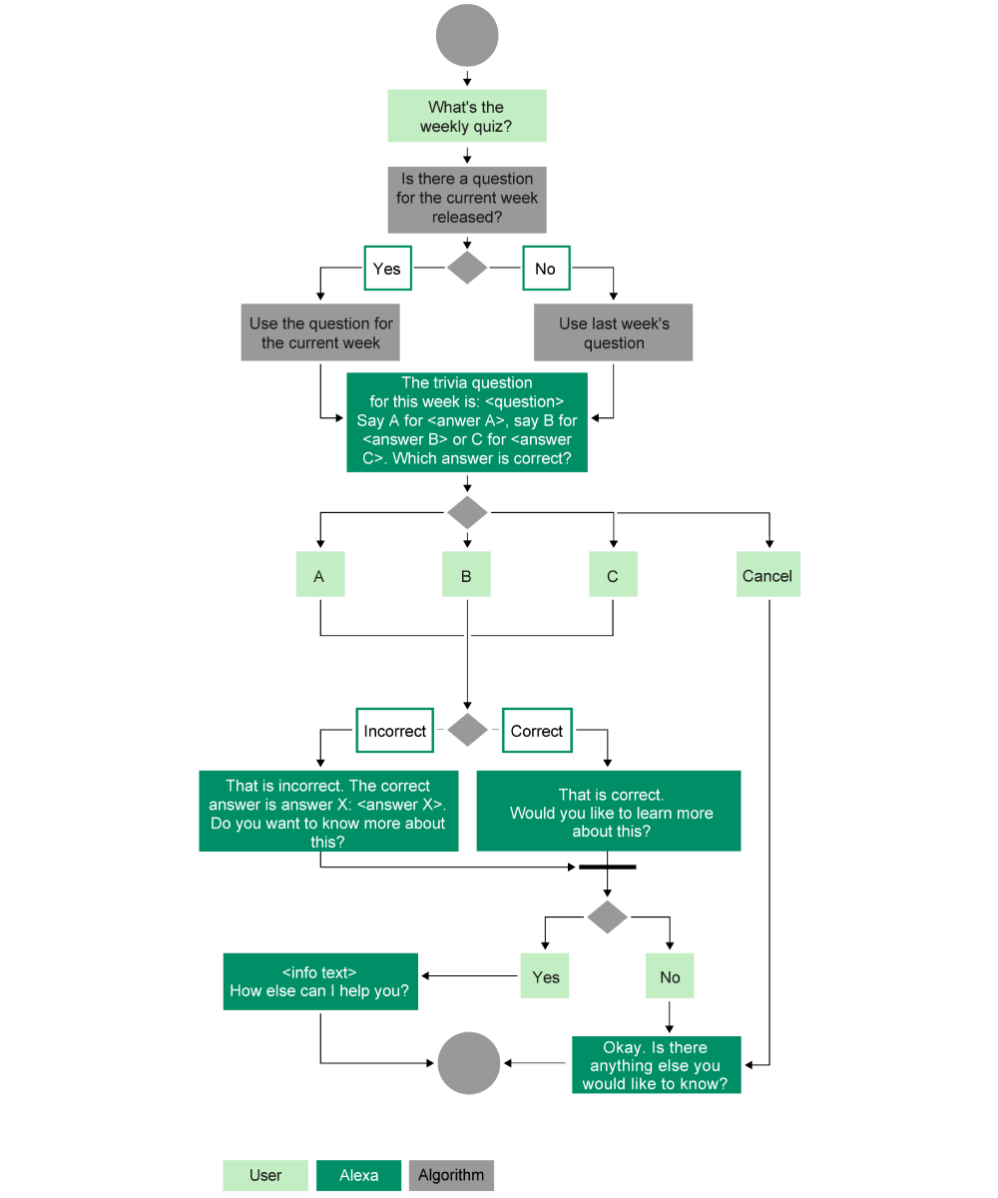
The algorithm of the multiple myeloma quiz functionality.

An interactive feature—an MM quiz functionality that provides multiple-choice questions that change on a weekly basis—was added to the skill. The quiz functionality can be actively started either by users asking for the quiz or by users waiting until they are asked if they want to answer the weekly quiz question after starting the skill. Alexa then provides the questions and 3 possible answers—“a,” “b,” and “c” (Multimedia Appendix 1). The users choose an answer and receive feedback on the answer. Thereafter, the skill offers the option for additional educational information (“Do you want to learn more on this?”). A sample question is shown in Figure 3. The quiz algorithm is shown in Figure 4.

### Usage Data

A total of 141 users (user enablements) have installed the skill since its launch (September 18, 2019, through July 1, 2021). In the time frame of the last 12 months (July 1, 2020, through July 1, 2021), a total of 189 skill sessions were retrospectively analyzed, which included 797 inquiries (utterances), indicating approximately 4 interactions per session. The most popular skill topics during this time frame were patient support groups near the users (58/797, 7.3%), information on MM (53/797, 6.6%), and the weekly quiz function (43/797, 5.4%). The average user retention rate in the analyzed time frame (3 users/week) decreased from 100% at its first use to 18.3% in week 1, 12.9% in week 2, and 1.3% in week 5. This was expectable, since we did not provide new content or news each week. Due to the limited number of total users and sessions in the analyzed time frame, the available usage data were not suitable for a more detailed analysis that would allow for useful interpretations.

The web-based survey on voice assistant usage and the feedback on the Alexa skill *Multiple Myeloma* were collected from 24 participants. These were patients and patient representatives (15/24, 63%), caregivers (6/24, 25%), HCPs (2/24, 8%), and other types of participants (1/24, 4%). Further, 54% (13/24) of the survey participants reported the prior usage of voice assistants (ie, mainly for listening to music or the radio and searching for information). Nonusers of voice assistants (11/24, 46%) stated that data protection concerns (7/11, 64%) and a lack of need (6/11, 55%) were the most important factors of not using voice assistants in general. Additionally, 50% (12/24) of participants had tested 1 or more functions of the Alexa skill *Multiple Myeloma*, and 46% (11/24) of participants would recommend the Alexa skill.

## Discussion

### Principal Findings

At MM diagnosis and during the MM disease course, patients with MM and their families are confronted with a lot of information and medical terms related to MM disease, diagnostics, and therapy, generating many questions. Often, patients and their families are not able to address their questions to an HCP directly when they occur. Hence, there is a need for patients and their families to have easy access to accurate, expert-reviewed information at the time they need it and in between formal appointments [6,8]. Further, patients with cancer are more vulnerable to the COVID-19 pandemic, which can impact their psychological health as well as their access to clinics [19,20]. As patient brochures or information on the internet might not be easily accessible for all patients and as older patients might have difficulties with reading or using a computer, voice assistants could offer a new opportunity to patients and their families. The Alexa skill *Multiple Myeloma* can be used with different devices, including smart speakers and smart TVs, and with smartphones by using the Amazon Alexa app with the integrated Alexa voice assistant. As all content of the *Multiple Myeloma* Alexa skill has been reviewed by cross-functional experts, the information is validated and is explained in patient-friendly language.

Although many questions can currently be answered by the skill, the content does not cover all possible questions, leaving room for future improvement. Furthermore, there are restrictions on content that pharmaceutical companies provide to patients based on laws and regulations in Germany; therefore, for example, no information on medications can be provided. The data on participants’ first use of the skill, although limited, show that the most popular skill features are directing patients to local patient support groups (58/797, 7.3%), followed by information on MM disease (53/797, 6.6%) and the weekly quiz function (43/797, 5.4%). The usage data, our patient survey, and personal feedback from medical experts and patient support groups have brought to light key challenges in using a medical voice assistant skill. As the median age of patients with MM at diagnosis is 69 years [1], this patient group may be difficult to reach via digital advertisement, and there are often technical obstacles that need to be overcome to install and use voice assistant skills. Therefore, we have added step-by-step instructions on how to use the skill for different devices, and our advertisements for the skill highlight the facts that no smart speaker is needed and that the skill can also be used with any smartphone that has the Alexa app. As we have identified data privacy concerns (7/11, 64%) as obstacles that limit more widespread adoption, especially since it was made public that an Amazon team listened to Alexa recordings to train its speech recognition and natural language understanding systems in 2019 [21], we further educated patients (via our advertisement of the skill) on questions regarding general data privacy concerns with voice assistants. We highlighted that users can change the Alexa privacy settings in the Alexa app if they do not want their voice recordings to be listened to by Amazon employees to improve Alexa’s services. Patient support groups suggested offering training to their local groups to introduce the skill and more effectively inform the patient populations in need of such a skill. Ideally, the initial installation and settings (including data privacy settings) are set up under the supervision of an experienced user (eg, in the context of patient support groups). Further limitations include the lack of experience with and use of voice assistants in daily life.

The speech recognition and interpretation of the questions are critical to the quality of the answers given by Alexa, even more so since specialized medical terms are being used. A failure to correctly understand or interpret the users’ questions was reported when using the skill. To improve these limitations, the skill’s content base could be expanded to cover more topics that are of interest to the users. Also, the addition of further utterances (wording variations of questions) to the existing intents would help users easily find their desired content. Generally, the more frequent use of the Alexa skill could improve its understanding and interpretation abilities through Amazon Alexa’s artificial intelligence engine. Further, training Alexa to respond to a user's voice can also contribute to the skill’s improvement.

Discussions about the future usage of Alexa skills and voice assistants for patients with MM among HCPs and patient organizations indicated that digital assistants and companions could be useful for promoting and supporting patients’ medication adherence. Proactive daily notifications, which can possibly be delivered via Alexa skill “routines,” for asking patients about their well-being, documenting side effects, and providing medication reminders could be useful additional features of an extended skill, thereby offering patients a more holistic “companion” during therapy. Medication adherence reminder systems for digital home assistants are currently being evaluated by investigators [22]. Side effects could possibly be documented in a diary function for patients’ next visit with a health care professional, and the voice assistant could ask patients to talk to their physician and offer to make a call if the algorithm detects side effects that should be looked at immediately. Functions that provide news on MM advancements in research and patient care could also be interesting features, especially if they can be made to adhere to data compliance and *Heilmittelwerbegesetz* regulations. Finally, voice assistants could also be useful in myeloma clinical studies, as they can be used to collect patient-reported outcomes via voice commands to make data collection easier and more comfortable for patients.

### Conclusions

The Alexa skill *Multiple Myeloma* answers frequently asked questions, explains medical terms, identifies nearby patient support groups, and includes a quiz with the goal of educating and empowering patients with MM.

## Appendix

Multimedia Appendix 1 Overview of the content in the Alexa skill "Multiple Myeloma" (eg, frequently asked questions and a quiz).

## Abbreviations

AMM: Arbeitsgemeinschaft Multiples Myelom
BMS: Bristol Myers Squibb
GSK: Glaxo Smith Kline
HCP: health care provider
LHRM: Leukaemiehilfe Rhein-Main
MM: multiple myeloma
NHS: National Health Service
TV: television
