# Supplementary material for: Convenient Access to Expert-Reviewed Health Information via an Alexa Voice Assistant Skill for Patients With Multiple Myeloma: Development Study
Source: JMIR Cancer. 2022 Jun 9;8(2):e35500. doi: 10.2196/35500 (PMC9227649; doi:10.2196/35500)
Supplement: Multimedia Appendix 1 [file cancer_v8i2e35500_app1.docx]

**Multimedia Appendix 1:**

**Content overview Alexa skill “Multiple Myeloma” (e.g. FAQ, quiz)**

| **Topic** | **Possible user questions** *(examples; variations possible)* | **Answer by Alexa skill “Multiple Myeloma”** *(English translation for illustration of this publication only)* |
| --- | --- | --- |
|  |  |  |
| **Multiple Myeloma disease, diagnostics and therapy** | | |
| **Multiple Myeloma** | | |
|  | - What is multiple myeloma?  - Tell me about multiple myeloma  - Explain the disease to me | Multiple myeloma is a cancer of cells of the body's immune system. The myeloma cells colonize the bone marrow diffusely and or in foci. Multiple myeloma is the most common bone or bone marrow cancer in Western countries. |
| **Myeloma Cells** | | |
|  | - What are abnormal plasma cells?  - Explain myeloma cells to me.  - What do you know about myeloma cells? | In multiple myeloma, the plasma cells are abnormal – this means that they are different from healthy cells. The abnormal plasma cells are also called myeloma cells. They divide uncontrollably and produce vast amounts of functionless antibodies even without a pathogen. Physicians refer to these antibodies as monoclonal or M proteins. |
| **Light chains** | | |
|  | -What are light chains?  - Explain to me what light chains are  - What do you know about light chains? | Light chains are a component of antibodies. There is a distinction between lambda and kappa light chains. In multiple myeloma, light chains are often produced in excess and are then measurable in the blood and urine with elevated levels. |
|  |  | *(optional additional information)* Some myeloma cells do not produce complete antibodies, but only light chains, in which case the disease is called light chain myeloma. |
| **Bones** | | |
|  | - What is osteolysis?  - Does multiple myeloma cause bone loss?  - Does multiple myeloma cause weak bones? | Myeloma cells growing in foci damage the structure of the bone marrow and lead to bone loss, also called osteolysis. |
|  |  | *(optional additional information)* It causes the bones to become unstable at these sites. They can break and there can be severe pain. In addition, the increased bone loss can disrupt the calcium balance in the blood. |
| **Symptoms** | | |
|  | - What are the symptoms of this disease?  - What are the symptoms?  - What are the effects of multiple myeloma? | Symptoms of multiple myeloma can vary widely, and include fatigue, anemia, bone pain, or frequent infections. The M-proteins and light chains produced by myeloma cells flood the body and clog the kidneys. This can lead to kidney damage. Due to the displacement of healthy hematopoietic cells in the bone marrow, not enough red blood cells can be produced. This leads to anemia, which can be manifested by fatigue and shortness of breath. In addition, too few white blood cells weaken the immune system, resulting in more frequent infections. Furthermore, myeloma cells growing in foci can damage the bones. |
| **Diagnosis** | | |
|  | - How is multiple myeloma diagnosed?  - Tell me about how the disease is diagnosed?  - What do you know about the diagnosis of multiple myeloma? | The symptoms of multiple myeloma can vary widely, and can include fatigue, anemia, bone pain, or frequent infections. Therefore, it is important for the physician to confirm a suspected diagnosis through various examinations.  Results of blood and urine tests, such as measurement of M protein, can provide clues to multiple myeloma. Usually, myeloma is then diagnosed by bone marrow biopsy. However, diagnostic imaging, such as X-rays of the bones, a computed tomography (CT) scan or a magnetic resonance imaging (MRI) scan, also help detect changes in bone structure and accumulations of myeloma cells. |
| **Treatment** | | |
|  | - Does myeloma always need treatment?  - When does multiple myeloma need to be treated?  - Is it mandatory to treat multiple myeloma? | Not all myeloma needs to be treated. Treatment of multiple myeloma is usually started only when there is a risk that the disease will lead to organ and kidney damage. This involves checking, for example, whether at least one of the so-called CRAB criteria is met - that is, whether there is elevated calcium in the blood, kidney dysfunction, anemia, or damaged bone. |
| **Therapy options** | | |
|  | - Tell me about the therapy options  - How is multiple myeloma treated?  - What are the treatment options? | There are a variety of treatment options available. The form of therapy depends on the stage of the disease, the genetic alteration and the patient's physical condition. For example, there is high-dose chemotherapy followed by  stem cell transplantation, chemotherapies and combination therapies with different drugs. These include proteasome inhibitors, immunomodulatory agents, monoclonal antibodies and HDAC inhibitors, for example. |
|  |  | *(optional additional information)* Proteasome inhibitors block the breakdown of proteins in myeloma cells, causing the myeloma cells to die. Immunomodulatory agents, among other mechanisms, stimulate the patient's own immune system against the myeloma cells. Monoclonal antibodies bind to specific structures of the myeloma cells and thus cause the cells to die via various mechanisms. HDAC inhibitors inhibit different HDAC-type enzymes, causing myeloma cells to die. |
| **Combination therapies** | | |
|  | - How does combination therapy work?  - Why are different drugs combined?  - What is the course of combined therapy? | To achieve the greatest possible effect against the tumor cells, combinations of different drugs that attack the myeloma cells at different sites are often used. These include for example proteasome inhibitors, immunomodulatory agents, monoclonal antibodies, and HDAC inhibitors. |
|  |  | *(optional additional information)* Proteasome inhibitors block the breakdown of proteins in myeloma cells, causing the myeloma cells to die. Immunomodulatory agents, among other mechanisms, stimulate the patient's own immune system against the myeloma cells. Monoclonal antibodies bind to specific structures of the myeloma cells and thus cause the cells to die via various mechanisms. HDAC inhibitors inhibit different HDAC-type enzymes, causing myeloma cells to die. |
| **Induction therapy** | | |
|  | - What is the process of induction therapy?  - What is induction therapy treatment?  - What is initial treatment? | With the help of induction therapy, multiple myeloma is first pushed back. After that, it can be decided whether consolidation therapy or maintenance therapy should still follow. Consolidation therapy is often high-dose chemotherapy followed by autologous transplantation of the patient's own cells. The goal of maintenance therapy is then to maintain the achieved state for as long as possible. |
| **Chemotherapy** | | |
|  | - What happens during high-dose chemotherapy?  - How are you treated during high-dose chemotherapy?  - What is high-dose chemotherapy? | High-dose chemotherapy followed by transplantation of the patient's own stem cells is today the standard therapy for patients in good general health, even at an older age. |
|  |  | *(optional additional information)* Autologous stem cell transplantation is the transplantation of the patient's own stem cells. High-dose chemotherapy followed by transplantation of the patient's own stem cells is today the standard therapy for patients in good general health, even at an older age. In order to destroy as many existing tumor cells as possible, a very high dose of chemotherapy is administered. This destroys the so-called hematopoietic bone marrow, including the healthy cells. Therefore, stem cells are taken from the patient's blood before starting the high-dose chemotherapy, then the high-dose chemotherapy is administered, and then the stem cells are returned after the treatment is completed. |
| **Stem Cell Transplantation** | | |
|  | - "What is s.c.t.?"  - "What does autologous stem cell transplantation mean?" | Autologous stem cell transplantation is the transplantation of the patient's own stem cells. High-dose chemotherapy followed by transplantation of the patient's own stem cells is today the standard therapy for patients with good general health, even at an older age. |
|  |  | *(optional additional information)* In order to destroy as many existing tumor cells as possible, a very high dose of chemotherapy is administered. This destroys the so-called hematopoietic bone marrow, including the healthy cells. That's why stem cells are taken from the patient's blood before high-dose chemotherapy begins, then the high-dose chemotherapy is administered, and then the stem cells are returned after the treatment is completed. |
| **Innovative Therapies** | | |
|  | - What other medications are available?  - What do you know about innovative therapies?  - What innovative therapies and approaches are there? | Intensive research has produced a number of new agents and innovative therapeutic approaches in recent years. These are now an important part of treatment and are often given in combination. These include, for example, proteasome inhibitors, immunomodulatory substances, monoclonal antibodies or HDAC inhibitors. But classical chemotherapies also continue to have a firm place in the treatment of multiple myeloma. |
|  |  | *(optional additional information)* Proteasome inhibitors block the breakdown of proteins in myeloma cells, causing the myeloma cells to die. Immunomodulatory agents, among other mechanisms, stimulate the patient's own immune system against the myeloma cells. Monoclonal antibodies bind to specific structures of the myeloma cells and thus cause the cells to die via various mechanisms. HDAC inhibitors inhibit different HDAC-type enzymes, causing the myeloma cells to die. Chemotherapy blocks cell division and thereby causes myeloma cells to die. |
| **Supporting treatments** | | |
|  | - What can be done against bone pain?  - What can I do to support therapy?  - What supportive treatments are available? | Supportive treatments, such as radiation therapy or taking bisphosphonates, are used to complement the treatment of multiple myeloma. |
|  |  | *(optional additional information)* Radiation therapy is used to selectively destroy myeloma foci in the bone with the goal of quickly relieving pain and preventing bone destruction. Bisphosphonates keep calcium in the bones and prevent bone breakdown. The goal is to stabilize the bones, provide pain relief and prevent fractures. |
| **Radiotherapy** | -"What do you know about radiotherapy?"  - "What do you know about radiation therapy?"  - "What happens during radiation therapy?" | Radiation therapy can be used together with the myeloma treatment. It involves the selective destruction of myeloma foci in the bone with the goal of rapid pain relief and prevention of bone destruction. |
| **Bisphosphonates** | -"What do you know about bisphosphonates?"  - "Bisphosphonates."  - "What are bisphosphonates?" | Bisphosphonates keep calcium in the bones and prevent bone breakdown. The goal is to stabilize bones, relieve pain, and prevent fractures. |
| **Relapse** | | |
|  | - "Will the disease come back?"  - "Does it relapse?"  - "Do relapses happen in multiple myeloma?" | Multiple myeloma is usually a progressive and recurrent disease. Despite intensive therapy after initial diagnosis, most patients experience a recurrence of the disease over time, known as a relapse. |
|  |  | *(optional additional information)* Today, there are a variety of modern treatment options available for relapse therapy that can at least temporarily control multiple myeloma. |
| **Cytogenetics** | | |
|  | - "What do you know about cytogenetics?"  - "Tell me about cytogenetics."  - "What is cytogenetics in multiple myeloma?" | Determining cytogenetics in multiple myeloma is important because myeloma cells can become more aggressive due to certain mutations. Typical examples in multiple myeloma are the translocation t(4/14) or the deletion del(17p). This may have an impact on choice of therapy. |
| **Translocation** | - "What does translocation mean?"  - "Explain translocation to me." | Translocation is the rearrangement of chromosomal segments, parts of the genetic material. This can activate genes that lead to cancer development. |
| **Deletion** | - "What does deletion mean?"  - "Explain deletion to me" | Deletion means that part of a chromosome, or genetic material, is lost. This can result in the loss of genes that would otherwise prevent cancer development. |
| **Amplification** | "What do you know about amplification?"  - "What is amplification?"  - "What does duplication mean?" | Amplification or gain means that parts of chromosomes or whole chromosomes are multiplied, for example, doubled. |
| **Further Information** | | |
|  | - "Where can I find more information?"  - "Who can I ask more questions?"  - "I have more questions" | For questions about the disease, patient support groups can also be a valuable contact. You can find information about regional groups and contacts, for example, via the Bundesverband Myelom Deutschland e.V. (www.myelom-deutschland.de), the Leukemia Aid Rhein-Main e.V. (www.lhrm.de), the AMM-Online (www.myelom.org) and the Deutsche Leukämie- & Lymphom-Hilfe (www.leukaemie-hilfe.de). |
| **Multiple myeloma related laboratory values** | | |
| **Blood tests** | | |
|  | - Why are blood tests important?  - Why should the blood be examined?  - Is a blood test important? | As part of the diagnosis of multiple myeloma and during long-term follow-up, various blood tests and laboratory tests are performed. These can provide information about the current condition, as well as about changes during the course of the disease. |
|  |  | *(optional additional information)* Blood cells are produced in the bone marrow. In multiple myeloma, there is an excessive proliferation of so-called plasma cells in the bone marrow. This can cause normal blood formation to be suppressed and not enough healthy blood cells to be produced. Therefore, careful monitoring of blood counts is important. |
| **Leukocytes** | | |
|  | -What are white blood cells?  -What are leukocytes?  -Why are white blood cells important? | White blood cells, also called leukocytes, are involved in fighting pathogens such as bacteria or viruses. If you don't have enough white blood cells, your body is not as capable of defending itself against infections and diseases. |
| **Erythrocytes** | | |
|  | - What are red blood cells?  - Why are red blood cells important?  - Tell me more about red blood cells | Red blood cells, also called erythrocytes, carry oxygen to the tissues of the body. Oxygen delivery to tissues depends on how many red blood cells you have and how well they function. Hemoglobin also plays an important role in this. |
| **Hemoglobin** | | |
|  | - What is hemoglobin?  - What does hemoglobin mean?  - What do low hemoglobin levels mean? | Hemoglobin is a protein in red blood cells that carries oxygen to all parts of the body. Low hemoglobin levels may be a sign that myeloma cells are preventing red blood cell production. Not having enough functioning red blood cells is also called anemia. This can make you feel tired and fatigued and short of breath. |
| **Neutrophils** | | |
|  | - What are neutrophils?  - What do you know about neutrophils?  - What are the different types of neutrophil granulocytes? | Neutrophils, or neutrophil granulocytes, are a special type of white blood cell that play an important role in fighting infections. Neutrophils are produced in the bone marrow and then released into the bloodstream, through which they travel to where they are needed. Because decreased numbers of neutrophils increase the risk of infection, their numbers must be carefully monitored during treatment. |
|  |  | *(optional additional information)* There are so-called rod-nucleated as well as segment-nucleated neutrophils. Rod-nucleated ones are precursors of segment-nucleated ones. |
| **Glomerular filtration rate** | | |
|  | - What is glomerular filtration rate?  - What does GFR mean?  - What does glomerular filtration rate mean? | Glomerular filtration rate (GFR) is a measure of kidney performance. A GFR that is too low may indicate impaired kidney function. Since renal dysfunction is often a consequence of myeloma disease, this value is checked regularly. |
| **Blood sugar** | | |
|  | - What is blood sugar?  - What is glucose?  - What is the role of blood glucose? | In a fasting state, glucose, also called blood sugar, is the most common type of sugar in the blood. The blood transports glucose to all the cells of the body, where it can then be used for energy production. |
| **Beta2-microglobulin** | | |
|  | - What is beta2-microglobulin?  - What is B2M?  - What does the beta2-microglobulin value tell you? | Beta2-microglobulin is a protein that is present in small amounts in the blood even in healthy people. In multiple myeloma, levels can be elevated. This protein does not cause problems itself, but it can serve as a useful indicator of prognosis i.e. a patient's outlook. High levels mean that the disease is more advanced. |
| **Serum protein electrophoresis** | | |
|  | - What does serum protein electrophoresis mean?  - What does SPE mean?  - What is an M gradient? | The body fights infections with antibodies called immunoglobulins (Ig) produced by plasma cells. In multiple myeloma, malignant plasma cells make an abnormal protein called M-protein or monoclonal protein. Serum protein electrophoresis can be used to separate the M protein in the blood from the other proteins and determine its amount. The M gradient is a large spike in the evaluation that indicates a large amount of M protein. The amounts of normal antibodies may also be decreasing. |
| **Patient support group search function** | | |
| **Group near the user** | - Is there a patient support group near me?  - Find a support group  - Where is the nearest patient support group? | - There is one group within 200 km. The group <name> is located in <city>." - There are two groups within 200 km. <name> is the closest and is located in <city>. The other group <name> is located in <city>. / <name> and <name> are both located in <city>.” - There are <number> groups within 200 km. <Name> is the closest and is located in <city >. // Or “<name> and <name> are both located in <city>” (additional information) The other groups are located in <city>, < city >, < city >, and < city >. - You can find a map of support groups and the exact addresses and information at [www.myelom-deutschland.de/selbsthilfegruppen](http://www.myelom-deutschland.de/selbsthilfegruppen). - Sorry, there is no group within 200 km. <name> is the closest group and is located in <city>/ |
| **Group in specific city** | - Is there a patient support group in Berlin? | According to Myelom Deutschland e.V., there is a patient support group in Berlin called Plasmocytoma/Multiple Myeloma. You can find the exact address and detailed information at www.myelomdeutschland.de/selbsthilfegruppen. |
| **Multiple myeloma quiz content** | | |
|  | **Quiz questions** | **Multiple choice answers (correct answer in bold)** |
|  | Myeloma cells are malignant abnormal plasma cells. What do healthy plasma cells do in the body? | **A - Normal plasma cells produce antibodies**  B - Normal plasma cells make blood plasma  C - Normal plasma cells build the bones |
|  |  | *(optional additional information)* Plasma cells are cells of the immune system. Normal plasma cells produce antibodies to defend the body against infections and release these into the blood. |
|  | What is the name of the protein that myeloma cells produce in high numbers? | A - X protein  B - Benno-Jim protein  **C - M protein** |
|  |  | *(optional additional information)* Myeloma cells produce large amounts of functionless antibodies called monoclonal protein, or M protein. |
|  | What is an autologous stem cell transplant? | A - A high-dose chemotherapy followed by a stem cell donation from an outside donor  B - A low-dose chemotherapy with vitamins  **C - A high-dose chemotherapy followed by administration of the patient's own stem cells** |
|  |  | *(optional additional information)* Autologous stem cell transplantation is the transplantation of the patient's own stem cells. This is today the standard therapy for patients with good general health, even at an advanced age. |
|  | What are light chains? | A) Complete immunoglobulins  **B) A component of immunoglobulins**  C) Chains of several immunoglobulins |
|  |  | *(optional additional information)* Light chains are a component of antibodies that are often produced in excess in multiple myeloma and can be measured in blood or urine. A distinction is made between lambda and kappa light chains. |
|  | Where are myeloma cells most common and can cause a lot of damage? | A - In the lungs  **B - In the bone marrow**  C - In the colon |
|  |  | *(optional additional information)* Myeloma cells grow in clusters in the bone marrow and can thereby both damage the structure of the bone and displace normal hematopoiesis. |
|  | What is osteolysis? | A) The drug-induced dissolution of a thrombosis  B) A therapy option in multiple myeloma for the treatment of bone pain  **C) The pathological breakdown of bone substance, e.g. in multiple myeloma** |
|  |  | *(optional additional information)* Myeloma cells grow in clusters in the bone marrow and can thereby both damage the structure of the bone and displace normal hematopoiesis. |
|  | Why do many myeloma patients have anemia? | A - This is purely coincidental  B - The myeloma cells feed on other blood cells  **C - The myeloma cells crowd out healthy hematopoiesis in the bone marrow** |
|  |  | *(optional additional information)* Anemia occurs when myeloma cells in the bone marrow displace normal blood formation and enough red blood cells can no longer be made. This can manifest as fatigue and shortness of breath. |
|  | What are hematopoietic stem cells? | a) Cells in the brain stem that play a role in poetic abilities.  b) Immature cells that begin to develop only after reaching puberty.  **c) Blood precursor cells located in the bone marrow that give rise to all blood cells.** |
|  |  | *(optional additional information)* Hematopoietic stem cells give rise to the normal blood components, including red blood cells, white blood cells, and platelets. For transplantation, these stem cells can be mobilized by stimulation and then be collected from the blood. |
|  | Which symptoms are typical for myeloma patients? | A - Yellowing of the skin  **B - Bone fractures and back pain**  C - Swollen lymph nodes |
|  |  | *(optional additional information)* Myeloma cells growing in the bone marrow can damage the bone and cause fractures. In addition, normal blood formation may be suppressed, leading to fatigue, anemia, or frequent infections. The kidneys can also be damaged by the abundant production of M-proteins and light chains |
|  | Which class of drugs is not one of the standard medications used in the treatment of multiple myeloma? | A - Immunomodulators  B - Proteasome inhibitors  **C - Antiviral drugs** |
|  |  | *(optional additional information)* Intensive research has produced a number of new innovative therapies in recent years. They are now an important part of treatment and are often given in combination with other drugs. These include for example proteasome inhibitors, immunomodulatory substances or monoclonal antibodies. However, classical chemotherapies also continue to have a strong role in the treatment of multiple myeloma. |
|  | What is the name of a precursor of multiple myeloma? | A) Heavy multiple myeloma  **B) Smoldering or smoldering multiple myeloma**  C) Leukemia |
|  |  | *(optional additional information)* Smoldering multiple myeloma is a precursor of multiple myeloma without symptoms that damage the body yet. |
|  | In younger or fitter patients, which therapeutic step usually begins first-line treatment? | **a. Induction therapy**  b. High-dose therapy and autologous stem cell transplantation  c. Maintenance therapy |
|  |  | *(optional additional information)* With the help of induction therapy, multiple myeloma is first suppressed in newly diagnosed younger or fitter patients. This can then be followed by high-dose chemotherapy with autologous stem cell transplantation, i.e. the patient's own blood stem cells. |
|  | Why do many myeloma patients have problems with their kidneys? | A - Myeloma patients do not drink much  **B - The myeloma cells produce large amounts of protein that can clog the kidneys**  C - It is just a coincidence |
|  |  | *(optional additional information)* The M-proteins and light chains produced by myeloma cells flood the body and clog the kidneys. This can lead to kidney damage. |
|  | What is anemia? | A) Decrease of white blood cells  **B) Decrease of red blood cells**  C) Decrease of blood platelets |
|  |  | *(optional additional information)* Anemia occurs when myeloma cells in the bone marrow displace normal hematopoiesis and the bone marrow can no longer make enough red blood cells. This can manifest as fatigue and shortness of breath. |
|  | What is meant by remission? | a) The repeated presentation to the hemato-oncologist.  b) The urge of a patient to talk a lot about his disease  **c) The decrease of signs of the disease** |
|  |  | *(optional additional information)* Remission is the term used by physicians to describe the reduction in the signs of disease. This is associated with a decrease in M protein and myeloma cells. |
|  | Which therapy is rarely used nowadays because of risks and side effects? | a. Autologous stem cell transplantation  **b. Allogeneic stem cell transplantation**  c. Antibody therapy |
|  |  | *(optional additional information)* Allogeneic stem cell transplantation is a transplantation of blood stem cells from a foreign donor. Due to possible undesirable immune reactions against healthy organs, this therapy is now used much less frequently in multiple myeloma than the transplantation of the patient's own stem cells |
|  | What is the name of the procedure for obtaining stem cells from blood? | **A) Leukapheresis**  B) Leukemia  C) Lymphatic drainage |
|  |  | *(optional additional information)* Leukapheresis is the technical term for the procedure to collect hematopoietic stem cells from the blood after appropriate stimulation. This is done, for example, before a stem cell transplant. |
|  | What are bisphosphonates? | A) Chemotherapeutic agent  B) Immunotherapy  **C) Group of substances for "bone protection”** |
|  |  | *(optional additional information)* Bisphosphonates keep calcium in the bones and prevent the breakdown of bone substance. The goal is to stabilize bones, relieve pain, and prevent fractures. |
|  | What does the term M-protein mean? | **A - Monoclonal protein produced by myeloma cells**  B - Mother protein, on which all proteins are based  C - Monovalent protein, a special form of protein that is uniformly shaped |
|  |  | *(optional additional information)* Myeloma cells produce large amounts of non-functional antibodies called monoclonal protein, or M-protein. |
|  | What does it mean when myeloma becomes refractory? | A - The treatment responds well  **B - The disease no longer responds to the therapy used**  C - The treatment has no side effects |
|  |  | *(optional additional information)* Refractory means that the disease is no longer responding to the current treatment. This may mean that the therapy needs to be changed. Today, there are a variety of modern treatment options available that can bring multiple myeloma under control, at least temporarily. |
|  | Why is the examination of cytogenetics important in multiple myeloma? | **A) The result of cytogenetics may have an impact on choice of treatment**  B) Cytogenetics helps the physician to know which therapy is guaranteed to work  C) This has always been done traditionally |
|  |  | *(optional additional information)* Determining cytogenetics in multiple myeloma is important because myeloma cells can become more aggressive due to certain changes in genetic material. This may have an impact on choice of therapy. |
|  | Is multiple myeloma hereditary? | A) Multiple myeloma is always inherited by the children.  B) This question has not been studied yet.  **C) Multiple myeloma is not a classic hereditary disease.** |
|  |  | *(optional additional information)* Multiple myeloma is not a classic hereditary disease. According to studies, the risk for first-degree relatives may nevertheless be slightly increased. However, due to the very low probability, it is not recommended to have relatives tested. |
|  | Why are regular blood tests important in multiple myeloma? | A) Blood values clearly show whether the patient is undergoing regular physiotherapy  **B) Blood values give the doctor important information about the current state and progression of the disease**  C) Myeloma cells can increase the production of healthy blood cells |
|  |  | *(optional additional information)* As part of the diagnosis of multiple myeloma and during long-term follow-up, blood tests and laboratory tests are performed to assess the current condition and changes in progression. In addition, careful monitoring is important because myeloma cells can suppress normal hematopoiesis. |
|  | What do myeloma experts do when a patient meets the so-called SLiM-CRAB criteria? | A) This does not affect therapy.  B) Therapy can be stopped.  **C) Myeloma therapy is started.** |
|  |  | *(optional additional information)* A few years ago, the so-called CRAB criteria were expanded to become the SLiM-CRAB criteria. Reaching these diagnostic criteria usually means the start of myeloma therapy for the patient. |
|  | Is there a difference between the terms multiple myeloma and plasmacytoma? | A) There is no difference  B) Yes, a plasmacytoma is benign, unlike multiple myeloma  **C) Yes, a solitary plasmacytoma, unlike multiple myeloma, is a rare collection of malignant abnormal plasma cells isolated in one location** |
|  |  | *(optional additional information)* A solitary plasmacytoma is a very rare, isolated collection of malignant abnormal plasma cells at only one isolated site - in contrast to collections at multiple sites in the bone marrow in multiple myeloma. Sometimes the term plasmacytoma is also used for multiple myeloma - but this is outdated. |
